# On Designing Multicore-Aware Simulators for Systems Biology Endowed with OnLine Statistics

**DOI:** 10.1155/2014/207041

**Published:** 2014-06-22

**Authors:** Marco Aldinucci, Cristina Calcagno, Mario Coppo, Ferruccio Damiani, Maurizio Drocco, Eva Sciacca, Salvatore Spinella, Massimo Torquati, Angelo Troina

**Affiliations:** ^1^Department of Computer Science, University of Torino, 10149 Torino, Italy; ^2^Department of Life Sciences and Systems Biology, University of Torino, 10123 Torino, Italy; ^3^Department of Computer Science, University of Pisa, 56127 Pisa, Italy

## Abstract

The paper arguments are on enabling methodologies for the design of a fully parallel, online, interactive tool aiming to support the bioinformatics scientists .In particular, the features of these methodologies, supported by the FastFlow parallel programming framework, are shown on a simulation tool to perform the modeling, the tuning, and the sensitivity analysis of stochastic biological models. A stochastic simulation needs thousands of independent simulation trajectories turning into big data that should be analysed by statistic and data mining tools. In the considered approach the two stages are pipelined in such a way that the simulation stage streams out the partial results of all simulation trajectories to the analysis stage that immediately produces a partial result. The simulation-analysis workflow is validated for performance and effectiveness of the online analysis in capturing biological systems behavior on a multicore platform and representative proof-of-concept biological systems. The exploited methodologies include pattern-based parallel programming and data streaming that provide key features to the software designers such as performance portability and efficient in-memory (big) data management and movement. Two paradigmatic classes of biological systems exhibiting multistable and oscillatory behavior are used as a testbed.

## 1. Introduction

This paper presents a critical rethinking of the parallelization of biological computational tools in the light of multicore platforms, which nowadays equip all scientific laboratories.

We will focus on the features that are required to derive an efficient simulator of stochastic processes considering, in particular, performance and easy engineering. This latter aspect will be of crucial importance for next generation of biological tools that will be largely designed by bioinformatics scientists, who are likely to be more interested in the accurate modeling of natural phenomena rather than on the synchronisation protocols required to build efficient tools on multicore platforms.

The stochastic simulation of biological systems is an increasingly popular technique in bioinformatics as either an alternative or a complementary tool to ordinary differential equations (ODEs). This trend, starting from Gillespie's seminal work [1], has been supported by a growing number of formalisms aiming to describe stochastic models of biological systems [2–7].

The stochastic modeling approach is computationally much more expensive than ODEs. Nevertheless, this approach is quite attractive for its superior ability to describe transient and multistable behaviors of biological systems. In particular it allows studying rare or divergent behaviors, spikes, and discriminate families of possible behaviors that are typically hidden in the averaged behavior described by ODEs.

The high computational cost of stochastic simulation can also be very annoying in the tuning of biochemical models in which some quantitative parameters are unknown or scarcely known and need a set of tests to be fixed.

This has led, in the last two decades, to a number of attempts to accelerate them up using several kind of techniques, such as approximate simulation algorithms and parallel computing [8, 9]. In this work, this latter approach is taken into account.

Since stochastic simulations rely on Monte Carlo method, many independent simulation instances should be computed and analysed to achieve statistically meaningful results. The computation of these independent instances has been traditionally exploited in an* embarrassingly parallel* fashion, executing a partition of the instances on different machines. This approach naturally couples with the distributed execution of a batch of tasks that require large infrastructures (e.g., grids and clouds) and suffers from slow time-to-solution as each experiment requires to enqueue the simulations in shared environment, deploy initial data, simulate the model, gather results from a distributed environment, postprocess them (often sequentially), then eventually access results. This process is typically repeated several times to fine tune the initial conditions and simulation parameters.

This approach, when transferred on multicore platform, which nowadays equips the large majority of computing platforms, fails shortly to produce actual application speedup, especially for I/O and memory-bound applications, since all the cores usually share the same memory and I/O subsystem. Indeed, the simulation of biological systems produces a large amount of data, which can be regarded as streams of data resulting from the on-going simulations. The management of these streams is not trivial on multicore platforms, as the memory bandwidth cannot usually sustain a continuous flux of data coming from all the cores at the same time. A related aspect concerns the filtering and the analysis of raw results, which require the merging of data obtained from different simulation instances, and possibly their statistical description or mining, with data reduction techniques. Even in a distributed computing environment this phase is often demoted to a secondary aspect in the computation and treated with offline postprocessing tools, frequently not even disclosed in performance results.

This approach is no longer practical, especially on multicore platforms, because of a number of reasons:

- the ever-increasing size of produced data burdens on the main weaknesses of multicore platforms, that is memory bandwidth and core synchronisations;
- the “sequentialisation” of simulation and analysis phases slow down the design-to-result process, which is particularly annoying during the tuning of the biological model, especially in the cases where the simulation outcome could show very soon an incorrect behavior;
- the design of the simulator is often specifically optimised for a specific parallel platform, either multicore or distributed (or not optimised at all).

Since the frequency and size of data moved across simulation workflows strictly depend on the required accuracy, the simulation and analysis of biological systems at high-precision happen to be a serious issue on modern shared-memory multicore platforms. Indeed it involves the merging of results from different simulation instances and possibly their statistical description or mining with data reduction techniques.

The design of simulation-analysis workflow encompassing a parallel simulator stage and a parallel data analysis stage is presented along with its experimental validation. The two stages are pipelined in such a way that, at each observed simulation time *t*_*i*_, the simulation stage stream out the partial results of all simulation trajectories (aligned at *t*_*i*_) to the analysis stage that immediately produces a partial result. Analysis stage, which can be equipped with user-defined statistic and mining operators, works on sliding data window and does not require keeping in memory the full data set with both performance and response time benefits.

The advocated design methodology is validated on interesting classes of biological problems, for which the classical modelization via ODE is quite problematic, if not impossible, while a stochastic model can be more proficuous. As we shall see, despite the whole simulation-analysis being performed on partial data, that is, on temporal sliding window of simulation results, the dynamics of the system is effectively approximated and can be shown to bioinformatics scientist during a simulation.

The technical challenges for the parallelisation of simulation and analysis stages and their pipelining are discussed (among these, the exploitation of high-level pattern-based parallel programming approaches to decrease design and implementation time). Eventually the proposed approach can be used as a fully reusable methodology that can be exploited in the design or parallelisation of other tools for systems biology.

The evaluation of the integrated approach will be focused on the efficiency and speedup of the tool in executing the simulation and online analysis workflow on multicore platforms. In this respect, paradigmatic examples of two challenging classes of biological systems, that is, bistable/multistable, and oscillatory systems, are discussed. The key behavior of these systems are represented by way of different classes of online analysis tools introduced in the previous section, respectively, statistical description, clustering, and frequency detection.

To perform these experiments, a simulator for the calculus of wrapped compartments (CWC), built with the above technology, will be used as test-bed. CWC [10] is a recently proposed formalism for the representation of biological systems. CWC extends the known stochastic simulators by adding a nested structure of labeled compartments delimited by membranes. However, to better focus on the proposed methodology and make the paper self-contained, we will write all examples of the paper in the basic subset of CWC in which biochemical reactions are denoted in a standard chemical notation. We only remark that a distinguished feature of CWC, which will be used in this paper, is the possibility of associating each reaction with an arbitrary rate function depending on the overall content of the ambient in which the reaction takes place. This allows to tailor the reaction rates on the specific characteristics of the system, as for instance when representing nonlinear reactions as Michaelis-Menten kinetics.

## 2. Methods

The methods for the stochastic simulation of biological systems, including Gillespie's algorithm [1], are typically based on the Monte Carlo method. An individual simulation, which tracks the state of the system at each time-step, is called* trajectory*. Many thousands of independent trajectories might be needed to get a representative picture of how the system behaves on the whole. This behavior springs from the collective analysis of trajectories, which is typically carried out by way of statistical or data mining estimators.

Thanks to their independence, the different instances needed to simulate a biological model that can be easily computed in an* embarrassingly parallel* fashion. However, the complete simulation workflow needed to derive simulation result including additional phases, such as the dispatching and scheduling of simulations, result gathering, trajectory data assembling, and analysis phases, which exhibit data dependencies (thus requires communications and/or synchronisations). Often, to simplify the design of the simulation tool, these phases are neither parallelized nor considered in the performance evaluation. As a matter of a fact, a parallel simulation is often considered an “embarrassingly parallel” problem, whereas it is if data distribution, gathering, filtering, and analysis are not considered as part of the whole simulation workflow. These phases, often (questionably) considered as preprocessing and postprocessing phases, may result to be as expensive as the simulation itself. Moreover, the full sequentialization of the phases inhibits the early detection of badly tuned simulations and makes the model tuning spiral annoyingly slow.

As an example, a simulation of the HIV diffusion problem (computed using the StochKit toolkit for 4 years of simulation time) may easily produce over 5 GBytes of raw data per instance [11]. As clear, the data size is *n*-folded when *n* instances are considered. Eventually, this data should be gathered and often reduced to a single trajectory via statistical methods or analysed with data mining methods, that can be much more time expensive to be figured out than bare statistical estimators.

These potential performance flaws are further exacerbated in multicore and many-core platforms. These platforms do not exhibit the same degree of replication of hardware resources that can be found in distributed environments and even independent processes actually compete for the same hardware resources within the single platform, such as main and secondary memory, the performances of which represent the real challenge of the forthcoming parallel programming models (a.k.a.* memory wall* problem). While simulation is substantially a CPU-bound problem on distributed platform, it may become prevalently an I/O-bound problem on a multicore platform due to the need to store and postprocess many trajectories. The finer the observed simulation time-step the strongest the computational problem is characterized as I/O-bound.

To be effective, stochastic methods in systems biology require many trajectories with a fine grain resolution in order to make observable deviant trajectories, peaks, high variance of results and multistable behaviors, which often represent the real nature of the phenomena that is not well captured by traditional approaches, such as ODEs. These events are often not immediate to detect in the bulk of gross simulation results. Several techniques for analyzing such data, for example, principal components analysis, linear modeling, and canonical correlation analysis have been proposed. It can be imagined that next generation software tools for natural sciences should be able to perform this kind of processing in pipeline with the running data source, as a partially or totally online process because:

- it will be needed to manage an ever-increasing amount of experimental data, either coming from measurement or simulation, and
- it will substantially improve the overall experimental workflow by providing the natural scientists with an almost real-time feedback, enabling the early tuning or sweeping of the experimental parameters and,thus, scientific productivity.

The flexibility given by the possibility of running many different analysis modules in parallel is of particular interest, as in many biological case studies the searched pattern in experimental results is unknown and might require to try different kinds of analysis since modules can be swept in parallel.

The parallel analysis of the system dynamics (e.g., along time) is more challenging since online data processing requires statistic and data mining operators to work on streamed data and, in general, random access to data is guaranteed only within a limited window of the whole dataset, while already accessed data can be only stored in synthesized form. When data description techniques, requiring to access the whole data set in random order, cannot be used, online data description and mining requires novel algorithms. The extensive study of these algorithms is an emerging topic in data discovery community and is beyond the scope of this work.

We advocate the parallelization of both simulations and analysis by pipelining them in a two-stage workflow, where both stages are also parallel. We also advocate the high-level design of the whole workflow to enhance both productivity and efficiency on different platforms (i.e., performance portability).

### 2.1. A Parallel Simulation-Analysis Workflow for CWC

The intrinsic complexity in the parallelization of the single step has traditionally led to the exploitation of parallelism in the computation of independent instances of the same simulation, which should anyway be computed to achieve statistical convergence of simulated trajectories (as in all Monte Carlo methods). The problem is well understood; it has been exploited in the last two decades in many different flavors and distributed computing environments, from clusters to grid to clouds [8, 11–16]. Notwithstanding that the problem has been often approached either neglecting to consider the cost of analysis or assuming that output data has a negligible size; this is not likely to happen in this and next generations biological simulations.

The previous considerations have led to the design of a simulation tool that includes both parallel simulation and data analysis in a single workflow. These phases are pipelined rather than sequential. To make it possible, it is needed that all the logical phases of the process (i.e., data distribution, parallel simulation result gathering, parallel trajectory data assembling, and analysis) can be effectively pipelined. This implies that all phases can effectively work on data streams. Ideally, an efficient and portable implementation of the simulation-analysis workflow should be able to represent streams as* first-class concept* and provide the designers with* high-level* programming constructs to manage them efficiently (also in multicore platforms).

To date, application programming for bioinformatics (and other sciences) has not embraced much more than low-level communication and synchronization libraries. In the hierarchy of abstractions, it is only slightly above toggling absolute binary in the front panel of the machine. The advent and the pervasiveness of multicore platforms are pushing parallel programming outside its historical niche. Next generation software should be designed not only to be* efficient* on these platforms, but also to be developed and tuned with* high-productivity* and reduced time-to-market. This is particularly important when parallel computing serves as a tool for other sciences since nonexpert designers should be able to experiment different algorithmic solutions for both simulations and data analysis.

Attempts to reduce the programming effort by raising the level of abstraction through the provision of parallel programming frameworks date back at least three decades and have resulted in a number of significant contributions. Notable among these is the* skeletal* approach [17] (a.k.a.* pattern-based* parallel programming), which appears to become increasingly popular after being revamped by several successful parallel programming frameworks [18–20].* Skeletons* (a.k.a. patterns) capture common parallel programming paradigms (e.g., Map, Reduce, MapReduce, Pipeline, Farm, and Divide&Conquer) and make them available to the programmer as high-level programming constructs equipped with well-defined functional and extrafunctional semantics [21]. Some of them are specifically designed to manage stream as first-class objects, such as* pipeline*,* farm* (a.k.a. master-worker pattern), and* loop* patterns.

**Figure 1:**
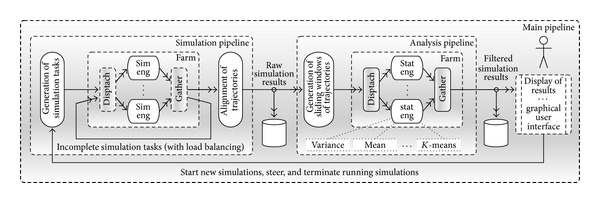
CWC simulator with online parallel analysis: architecture.

A particularly efficient implementation of the described patterns is provided by the FastFlow parallel programming framework [22]. FastFlow is a C++ open-source template library aimed at simplifying the development of efficient applications for multicore platforms. FastFlow eases the development and guaranteed runtime efficiency by raising the abstraction level of the design phase, thus providing developers with a set of optimised parallel programming patterns [22, 23]. Run-time efficiency is mainly achieved by way of a lock-less implementation of patterns exhibiting a speed edge against other popular parallel programming frameworks (also see performance comparisons in [24]). The FastFlow implementation of the pipeline, loop, and farm patterns is exploited to design the CWC simulation workflow. The effectiveness of the FastFlow framework in high-frequency synchronizations (i.e., fine-grained tasks) at a high-level of abstraction is the key features to devise a portable and efficient implementation of the simulation workflow, which is CWC simulator with online parallel analysis: architecture, composed by a three-stage pipeline: simulation, analysis, and display of results. The former two stages are in turn pipelines of other stages, whereas the display of results is realized by way of a graphical user interface (GUI). The big picture of the simulation workflow is shown in Figure 1. In the picture, all the grey boxes as well as all the code needed for synchronization and data streaming (double-headed arrows) are automatically generated by the FastFlow framework. The implementation of the whole software actually consists in declaring the structure of the workflow in terms of FastFlow objects (i.e., farm and pipelines) and filling white boxes with sequential code. All data is passed by using their references, in such a way that no data copies in memory are needed.

#### 2.1.1. The Simulation Pipeline

The simulation pipeline, as shown in Figure 1, is composed by three main stages: (i)* generation of simulation tasks*, (ii)* farm of simulation engines,* and (iii)* alignment of trajectories*.

The input of the simulation pipeline (from the GUI or a file) is the model to be simulated and the parameters of the simulation. The output is a stream of simulation results, each of them being an array holding a point for each of the trajectories of all (independent) simulations aligned at a given simulation time. Actually, each array represents a cut at a given simulation time of the whole dataset of results. This does not necessarily represent the current status (at a given point in wall-clock time) of all running simulations. By their very nature, stochastic processes exhibit an irregular behavior in space and time, since different simulations may cover the same simulation timespan following many different, randomly chosen, paths and number of iterations. Therefore, parallelization tools should support the dynamic and active balancing of workload across the involved cores. This mainly motivates the structure of the simulation pipeline. The first stage generates a number of independent simulation tasks, each of them wrapped in a C++ object. These objects are passed to the farm of simulation engines, which dispatch them (on-demand) to a number of simulation engines (sim eng). Each simulation engine brings forward a simulation for a given simulation time (simulation quantum) then reschedules back the simulation along the feedback channel. Simulation results produced in this quantum are streamed toward the next stage which sorts out all the received results and aligns them according to simulation time. Once all simulation tasks overcome a given simulation time, an array of results is produced and streamed to the analysis pipeline.

In this process, the farm schedule prioritizes “slow” simulation tasks, in such a way that the simulation tasks proceed with simulation times the more aligned as possible. This solves the load balancing problem by keeping all simulation engines always busy and reduces to the minimum the transient storage of incomplete results, thus reducing the shared memory traffic.

#### 2.1.2. The Analysis Pipeline

By design, each snapshot at a given simulation time of all simulation trajectories (i.e., an array of simulation results) can be analyzed immediately and independently (thus concurrently) on each other. For example, the mean and variance (as well as other statistical estimators) can be immediately computed and streamed out to the display stage. More complex analyses, that is, the ones aimed to understand system dynamics, have further requirements. In the most general case, they require the access to the whole dataset. Unfortunately this can be hardly done with a fully online process. In many cases, however, it is possible to derive reasonable approximations of these analyses from a sliding window of the whole dataset (e.g., for trajectory clustering). For this, the stream incoming in the analysis pipeline is passed through a stage that creates a stream of (partially overlapping) sliding windows of trajectories cuts. Each sliding window can be eventually processed in parallel and therefore is dispatched to a farm of statistic engines. Results are collected and reordered (i.e., gathered) and streamed toward the user interface and the permanent storage.

The analysis pipeline is provided with three families of predefined estimators covering the most common instruments of statistical analyses. Thanks to high-level modular design of the simulation pipeline, it can be easily extended with new filters. Current filters included in the system are as follows.

(*1) Mean, Standard Deviation, and Quantiles*. These standard statistical estimators are typically used to evaluate, both qualitatively and quantitatively, the behavior of stable systems and the reliability of the stochastic models used for their simulation. Quantiles are also often useful to approximate the distribution of simulation trajectories over time as it performs a histogram which summarizes the involved quantities without the effects of long-tailored asymmetric distribution or outliers. In fact, in those cases, descriptive statistics could not underline a central tendency.

(*2) Trajectory Clustering*. The clustering of trajectories helps the analysis of biological systems exhibiting a multistable behavior. Each cluster can automatically separate and distinguish different cases which can be eventually analyzed by statistical estimators. Concentrations of elements, in a given instant, from all simulations, are numerically filtered from stochastic noise and the global trends are extrapolated from clusters. In this work we employed two clustering techniques:* K*-means [25] and quality threshold (QT) [26] clustering. The clustering procedure collects the filtered data contained into a sliding time window Δ_*W*_ centered in the current data point *x*_*i*_ ≡ *f*(*t*_*i*_) where *t*_*i*_ ≡ *t*_0_ + *i*Δ_*S*_ (where Δ_*S*_ is a constant sampling time) for all simulation trajectories and the extrapolated forecast point, *x*_*i*_^*E*^, referred to the local trend, using the information of the Savitzky-Golay filter [27], that is, a low-pass filter suitable for data smoothing. The main idea underneath Savitzky-Golay filtering is to find filter coefficients *c*_*n*_ that preserve higher moments, that is, to approximate the underlying function within the moving window not by a constant (whose estimate is the average) but by a polynomial of higher order. This schema also allows the computation of numerical derivatives considering the coefficient of the derived polynomial.

(*3) Peak/Frequency Detection*. Many processes in living organisms are oscillatory. For these kinds of systems the analysis must be focused on the recurrence of phenomena, for instance concentration spikes or peaks of biological quantities, which also make it possible to determine the frequency of occurrence of a given phenomenon. The peak detection is basically performed by way of the analysis of the local maximum in a continuous curve, which is in turn detected through the analysis of the derivatives of the curve estimated by the Savitzky-Golay filter. From the period between successive peaks, the frequency of the related event is then inferred.

The usage examples of each family of filters will be discussed in the “results” section.

#### 2.1.3. The Graphical User Interface

**Figure 2:**
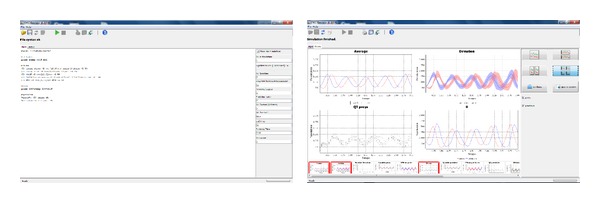
Screenshots of the simulation tool interface.

The CWC simulation-analysis pipeline is wrapped in a back-end tool that can be steered either via a command line tool or via a graphical user interface. This makes it possible to design the biological model, to run simulations and analysis, and to view partial results during the run. Also, the front-end makes it possible to control the simulation workflow from a remote machine. Two screenshots of the graphical front-end are reported in Figure 2.

## 3. Experiments and Results

The evaluation of the integrated approach will be focused on the efficiency and speedup of the tool in executing the simulation and online analysis workflow on multicore platforms. In this respect, paradigmatic examples of two challenging classes of biological systems are discussed, that is bistable/multistable, and oscillatory systems. The key behavior of these systems is studied by way of the different classes of online analysis tools introduced in the previous section, in particular, statistical description, clustering, and peak/frequency detection.

### 3.1. Expressivity and Effectiveness

#### 3.1.1. Multistable Biological System (Schlögl Model)

One of the most studied examples of bistability is the Schlögl model [28]. The simplicity of this network makes it an ideal prototype to show the effectiveness of the online clustering techniques on the filtered trajectories in the presence of bimodality. The set of reaction rules modeling this system are

(1) $$\begin{array}{c} A\;A\overset{0.03}{⟼}A\;A\;A\;\;A\;A\;A\overset{0.0001}{⟼}A\;A\\ B\overset{200}{⟼}B\;A\;\;A\overset{3.5}{⟼}•, \end{array}$$

where the rules are decorated with the kinetic constants of the corresponding reactions and all reaction rates are evaluated according to the mass action law.

**Figure 3:**
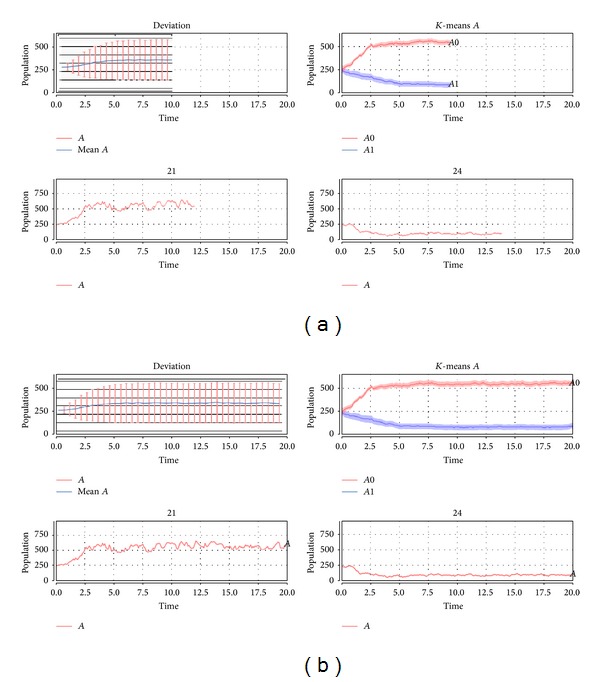
Simulation results on the Schlögl model. The figures report the mean and standard deviation, two exemplificative raw simulation trajectories, and the clustering results using* K*-means during the simulation runs (a) and at the end of the simulation runs (b).

The number of molecules of the species *B* is kept constant (buffered), while at equilibrium, the species *A* displays a noise-induced switching between the two stable steady states (see Figure 3). This case is paradigmatic to show that simple mean and standard deviation are not significant to summarize the overall behavior and the mean is not representative of any simulation trajectory.

Figure 3 shows the resulting clusters computed online using* K*-means on the Schlögl model for species *A* over 480 stochastic simulations starting with the term *B*  250∗*A*. The lines width of the* K*-means plot is proportional to each cluster size.

#### 3.1.2. Multistable System (Bacteriophage *λ* Life Cycle)

**Figure 4:**
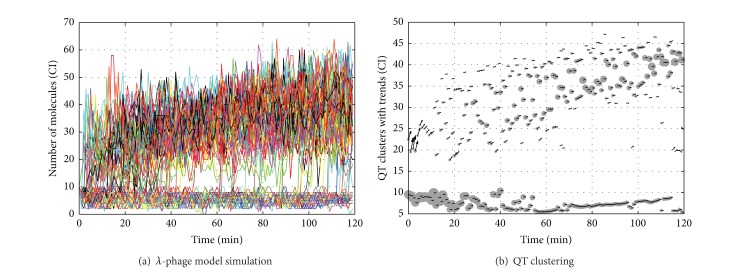
Simulation results on the *λ*-phage model. (a) Reports (approximatively) the 480 raw trajectories and (b) shows the online clustering results using QT.

One of the best studied examples of multistability in genetic systems is the bacteriophage *λ* life cycle [29]. This process involves two different biological entities delimited by membranes, the phage, and the bacterium. Lambda phage is a virus consisting of a head, containing a double-stranded linear DNA and a tail. The phage recognizes and binds to its host,* Escherichia Coli*, causing the DNA in the head of the phage to be ejected through the tail into the cytoplasm of the bacterial cell. After this, it can enter into one of two alternative stages called lysogeny and lysis. The lysogenic stage is a dormant stage in which the phage DNA is inserted into the host DNA and passively reproduces with the host. The only protein expressed in this phase is the *λ* repressor *CI*. When the host becomes stressed, the phage is more likely to go into lysis, in which case it reproduces more phages, kills the host, and spreads to other bacteria. The decision between lysis and lysogeny can be thought of as a switching mechanism. A simplified model for the bacteriophage was proposed in [30]. In their model, the gene cI expresses the *λ* repressor (*CI*) which dimerises (*CI*2) and binds to DNA (*D*) as a transcription factor at either of the two binding sites. The binding of the transcription factor to the site enhancing the transcription of *CI* (positive feedback) is represented by *D* ^+^ *CI*2. The phagic DNA in state *D* ^+^ *CI*2 leads the lysogenic stage. The binding of the transcription factor to the site repressing the transcription of *CI* (negative feedback) is denoted by *D* ^−^ *CI*2. The notation *D* ^+^ *CI*2*D* ^−^ *CI*2 models the phagic DNA when both sites are bound (*CI*2 can bind to the repressing site also when another *CI*2 dimer is bound to the promoting site, with a global repressing effect). The reaction rules in this system are

(2) $$\begin{array}{c} CI\;CI\overset{0.05}{⟼}CI2\;\;CI2\overset{0.5}{⟼}CI\;CI\\ CI2\;D\overset{0.026}{⟼}D^{+}CI2\;\;D^{+}CI2\overset{0.026}{⟼}CI2\;D\\ CI2\;D\overset{0.026}{⟼}D^{-}CI2\;\;D^{-}CI2\overset{0.026}{⟼}CI2\;D\\ D^{+}CI2\;CI2\overset{0.13}{⟼}D^{+}CI2D^{-}CI2\\ D^{+}CI2D^{-}CI2\overset{0.13}{⟼}D^{+}CI2\;CI2\\ D^{+}CI2\;P\overset{40}{⟼}D^{+}CI2\;P\;CI2\;CI2\;\;CI\overset{0.0007}{⟼}•, \end{array}$$

where *P* represents the RNA polymerase, assumed here to be constant, and two proteins per mRNA transcript were considered. In this model, the stochastic time trajectories of *CI* switch between two stable equilibria if the noise amplitude is sufficient to drive the trajectories occasionally out of the basin of attraction of one equilibrium into the basin of attraction of the other equilibrium (see Figure 4(a)).

Figure 4(b) shows the resulting clusters (gray circles) computed online using QT on the *λ*-phage model for species *CI* over 1200 stochastic simulations starting with the term 10∗*CI* *D* *P*. Circles diameters are proportional to each cluster size and arrows display the local trends of the clustered trajectories.

*K*-means is suitable for stable switch systems where the number of clusters and their tendencies are known in advance, in the other cases QT, although more computationally expensive, can build accurate partitions of trajectories giving evidence of instabilities with a dynamic number of clusters.

#### 3.1.3. Oscillatory System (Circadian Oscillations of Neurospora)

We examine here the theoretical model for circadian oscillations based on transcriptional regulation of the frequency (frq) gene in the fungus* Neurospora*. The model relies on the feedback exerted on the expression of the frq gene by its protein product FRQ · FRQ_in_ represents the FRQ protein inside the nucleus. In this model, sustained rhythmic variations in protein and mRNA (*M*) levels occur in the form of limit cycle oscillations [31]. In describing this system we exploit a feature of CWC which allows computing the rate of some reaction with an ad hoc function used to represent nonstandard kinetics. The reaction rules modeling this case are

(3) $$\begin{array}{c} \text{FR}{\text{Q}}_{\text{in}}\overset{f_{\text{FR}{\text{Q}}_{\text{in}}(t)}}{⟼}\text{FR}{\text{Q}}_{\text{in}}\;M\\ M\overset{0.5}{⟼}M\;\text{FRQ}\\ M\overset{f_{M}}{⟼}•\;\;⊤:\text{FRQ}\overset{f_{d}}{⟼}•\\ \text{FRQ}\overset{0.5}{⟼}\text{FR}{\text{Q}}_{\text{in}}\\ \text{FR}{\text{Q}}_{\text{in}}\;\overset{0.6}{⟼}\text{FRQ}. \end{array}$$

The model is based on the negative feedback exerted by the protein FRQ on the transcription of the frq gene; the rate of gene expression is enhanced by light. The model includes gene transcription in the nucleus, accumulation of the corresponding mRNA in the cytosol with the associated protein synthesis, protein transport into and out of the nucleus, and regulation of gene expression by the nuclear form of the FRQ protein. The function *f*_FRQ_(*t*) = *v*_*s*_(*t*)(*K*_*I*_^*n*^/(#FRQ^*n*^ + *K*_*I*_^*n*^)) denotes the rate of frq transcription where #FRQ denotes the number of FRQ elements at the moment in which the reaction takes place. The parameter *v*_*s*_(*t*) defined by:

(4) vs(t)={160 when  2nT≤t<(2n+1)T200 when  (2n+1)T≤t<(2n+2)T(n≥0)

increases in light conditions of the current time of the simulation, where *T* represents the period of the dark-light phases. The constant *K*_*I*_ is related to the threshold beyond which nuclear FRQ represses frq transcription; the Hill coefficient, *n*, characterizes the degree of cooperativity of the repression process. In the functions, the name of an atom indicates its multiplicity. The mRNA degradation is given by the Michaelis rate function *f*_*M*_ = *v*_*m*_(#*M*/(*K*_*M*_ + #*M*)). The FRQ degradation is given by the Michaelis rate function *f*_*d*_ = *v*_*d*_(#FRQ/(*K*_*d*_ + #FRQ)), where *v*_*d*_ is the maximum rate of FRQ degradation and the Michaelis constant related to this process is *K*_*d*_.

**Figure 5:**
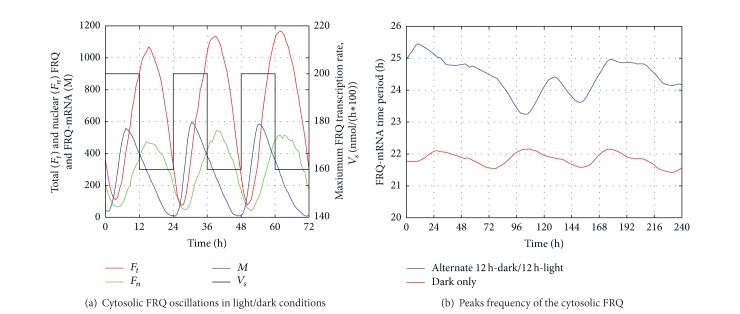
Simulation results of the cytosolic FRQ protein of the* Neurospora* model.

As in [31] we modeled the oscillations under two different conditions: (i) constant dark conditionand (ii) alternate light and dark phases. Following [31], the values of the parameters are set as *v*_*m*_ = 50.5, *v*_*d*_ = 140, *k*_*s*_ = 0.5, *k*_1_ = 0.5, *k*_2_ = 0.6, *K*_*m*_ = 50, *K*_*I*_ = 100, *K*_*d*_ = 13, and *n* = 4. Concentrations have been made discrete by scaling 1 nM to 100 atomic elements. In the constant dark condition, parameter *v*_*s*_ is equal to 160, in the alternate condition, *v*_*s*_ is equal to 160 during the dark phase and to 200 during the light phase.  Figure 5(a) shows an extract of a single stochastic simulation of the circadian oscillations in the dark/light alternate condition, plotting the number of FRQ proteins within the nucleus, the total number of FRQ proteins in the cell and the number of mRNA molecules leading the synthesis of FRQ.  Figure 5(b) shows the outcome of the peak detection tool which is able to summarize the frequency of the peak events over time. The plot results after capturing the peaks in the curve of the cytosolic mRNA for the FRQ protein synthesis. Measuring the distance between two consecutive peaks, we compute the period of each oscillation and then plot the moving average, over 5000 simulations, of the local periods. In the constant dark condition, the circadian period is close to 21 and a half hours, but increases, producing damping oscillations with a period of approximately 24 hours, in the dark/light alternate condition.

### 3.2. Performances

All reported experiments were run on an Intel workstation equipped with 4 eight-core E7-4820 Nehalem (32 cores, 64 contexts) @2.0 GHz with 18 MB L3 cache and 64 GBytes of main memory with Linux x86_64.

The analysis pipeline is configured with 3 statistic engines executing mean, standard deviation, quantiles,* K*-means, QT, and frequency detection filters. For each experiment the total number of FastFlow nodes, that is, the boxes depicted with solid lines in Figure 1, is

(5) #(sim  eng)+#(stat  eng)+#(other  nodes) +#(FastFlow  support  nodes)=#(sim  eng)+3+3+4,

where other nodes are “generation if simulation tasks,” “alignment of trajectories,” and “generation if sliding windows of trajectories” nodes, whereas FastFlow support nodes are the two couples of dispatch-gather nodes in Figure 1. Each node in the FastFlow run-time support is implemented by a POSIX (portable operating system interface for uniX) thread using a nonblocking execution model.

As we shall see, the number of statistical engines has been chosen according to a simple but effective performance model, which is made possible by the high-level approach of the design. According to the same model, the most interesting sensitivity analysis under performance viewpoint concerns the number of simulation engines.

As a case study, we consider the simulation workflow for the transcriptional regulation of the Neurospora.

**Figure 6:**
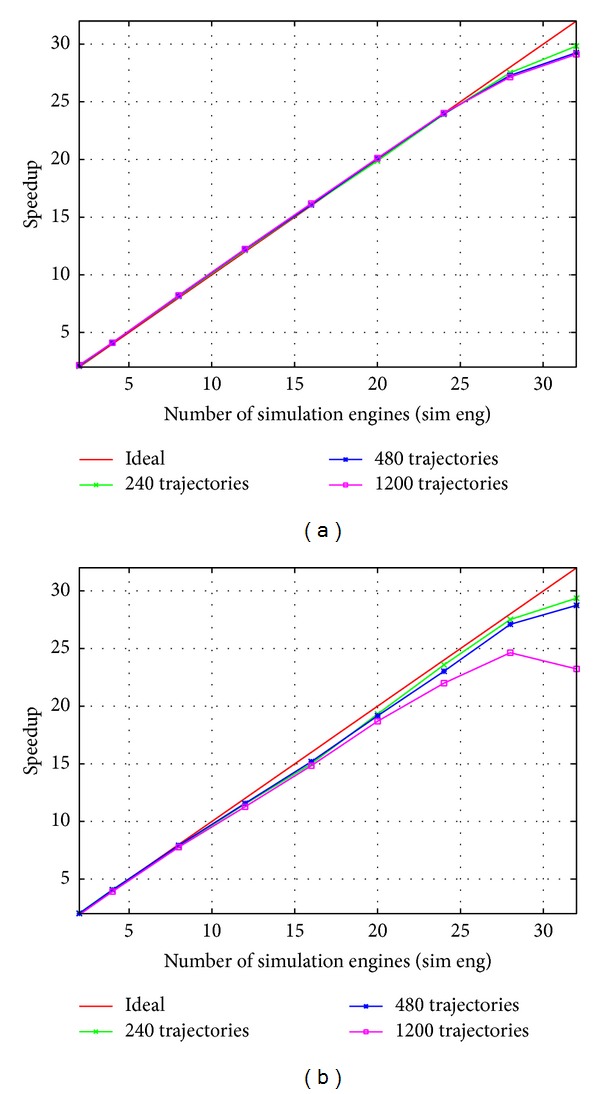
Speedup of the workflow of the Neurospora model on the Intel platform against number of simulation engines with 3 statistical engines, for different number of trajectories, each of them counting 10^4^ points (a) and 10^5^ points (b).

Figure 6 shows the speedup obtained for the whole workflow on varying the number of concurrent simulation engines, where the simulation points (or* samples*) per trajectory is set to be 10^4^ and 10^6^ simulation points.

The speedup on the total execution time achieved in the former case (Figure 6(a)) scales ideally with respect to the number of simulation engines, whereas a performance penalty is paid in the latter case (Figure 6(b)) for the highest degree of parallelism and number of produced trajectories.

The very same speedup behaviour is achieved for other test cases and it is worth a detailed discussion. For each performance experiment all the runs are executed by fixing random seeds. Thus, given a set of simulation parameters, it can be verified that each stochastic simulation of a single trajectory requires exactly the same number of iterations and the simulated time progress identically across random walks irrespectively of the number of simulation/statistic engines and* observed* simulation points, which can be considered a (synchronized) sampling at fixed simulation times of trajectories. These observations imply the following.

- The parallelism strategy does not break determinism and reproducibility of results (correctness).
- As reflected in the speedup results, the design of the simulator ensures effective load balancing and low synchronization overheads.
- The efficiency of parallel executions depends on the order of magnitude of the observed simulation points and by the number of produced trajectories.

This latter point specifically exploits the working hypothesis: stochastic methods are particularly informative when used to simulate the model at high resolution, that is, high number of samples and trajectories. In this case, the main bottleneck of the simulation software is data movement and management since the computation/data-movement ratio may easily reach the limits of modern multicore platforms.

In multicore platforms, “observing” the phenomena is a key issue in the simulation-analysis workflow as the frequency of observation determines both the quality of results and, inversely, the overall speedup. As shown in Figure 6 and Table 1, the proposed design and implementation effectively cope with this trade-off and succeed to exploit high rates of data movement. Thanks to merging many independent trajectories happening in the simulation pipeline, the output size, and, thus, the required disk throughput is greatly reduced (unless the storage of raw simulation results, happening among the two pipelines, is requested by the user).

The proposed simulation architecture is not only fast but also highly predictable in term of performance. This latter aspect is mainly due to the high-level structured design [32]. The whole workflow is a pipeline of two pipelines (i.e., a pipeline), whose performance can be modeled by means of the* service time *(*Ts*) of each stage *S*_*i*_. In particular

(6) $$\begin{array}{c} Ts\left(pipeline\left(S_{1},\ldots S_{k}\right)\right)=\max\left\{Ts\left(S_{1}\right),\ldots Ts\left(S_{k}\right)\right\}, \end{array}$$

where *Ts*(*S*_*i*_) models the average interdeparture time of stream items of the stage *i* of the pipeline, which actually matches the average computation time of *S*_*i*_ to produce one stream item. In turn, some of the stages are farm, which exploit *n* independent replicas of a (sequential or parallel) worker, for example simulation and static engines. Its service time can be modeled as

(7) $$\begin{array}{c} Ts\left(farm\left(W,n\right)\right)=\frac{Ts\left(W\right)}{n}. \end{array}$$

Given the service time of each sequential stage, for example, measured in the sequential code, these equations can be also used to tune the optimal number of workers *n* for any new simulation problem and to understand its upper bound in term of speedup. As an example, in the Neurospora with 10^5^ samples test case the sequential code exhibits the following timing per trajectory: *Ts*(generation)~0, *Ts*(sim eng) = 5.3 s, *Ts*(alignment) = 0.11 s, *Ts*(windows generation) = 0.02 s, and *Ts*(stat eng) = 0.33 s, with a total execution time for each trajectory of ~5.8 s (~120 minutes for 1200 trajectories). Among those, sim eng and stat eng are used within a farm, thus their service time can be reduced by increasing the number of workers. Therefore, the maximum performance and efficiency of the whole workflow are reached when the two farms are tuned to match the service time of the slowest sequential stage, that is, the alignment stage 0.11 s. For this, the farm in the simulation pipeline should be configured with *n* = 5.3/0.11 ≈ 48 workers, whereas the farm in the analysis pipeline with *n* = 0.33/0.11 = 3 workers. The overall speedup upper bound can be obtained using the total execution time and the slowest stage service time, that is, maximum speedup achievable for this test case is *≈*5.8/0.11 = 53, which includes the contributes from both pipeline and farm. The analysis, despite being approximated since it does not include synchronization overheads and memory bandwidth limits, is adherent of results depicted in the left plot of Figure 6. The speedup linearly grows with the number of simulation engines in the *n* = [1 ⋯ 32] range. The primary reasons of the slight performance drop in the right end of the plots is due to the fact that more virtual cores (i.e., hyperthread contexts) than physical core are used and the increased memory traffic for high numbers of trajectories. Furthermore, the performance analysis highlights that the bottleneck of the architecture for high throughput problems, which is in the alignment of trajectory stage. Its parallelization, which can be addressed by pipelining simulation engines and a partial alignment stage within the farm, is among future works.

However, this simple reasoning does not apply when a big number of trajectories are modeled. In fact, in such cases, the main architecture bottleneck when using a high number of simulation engines is the memory bandwidth limit of the underlying platform. Such effect can be seen in the right plot of Figure 6 for the case of 1200 trajectories.

## 4. Discussion and Related Works

In the field of biological modeling, tools such as SPiM [33, 34] and Dizzy [35] have been used to capture first order approximations to system dynamics using a combination of stochastic simulation and differential equation approximation. SPiM has long been the standard tool for simulating stochastic *π* calculus models.

Bio-PEPA [36] is a timed process algebra designed for the description of biological phenomena and their analysis through quantitative methods such as stochastic simulation and probabilistic model-checking. Two software tools are available for modeling with Bio-PEPA: the Bio-PEPA Workbench and the Bio-PEPA Eclipse Plugin [37].

The parallelization of stochastic simulators has been extensively studied in the last two decades. Many of these efforts focus on distributed architectures. Our work differs from these efforts in three aspects (see discussion below): (1) it addresses multicore-specific parallelization issues; (2) it advocates a general parallelization schema rather than a specific simulator, and (3) it addresses the online data analysis, thus it is designed to manage large streams of data. To the best of our knowledge, many related works cover some of these aspects, but few of them address all three aspects.

The Swarm algorithm [38], which is well suited for biochemical pathway optimisation, has been used in a distributed environment. An example is Grid Cellware [39], a grid-based modeling and simulation tool for the analysis of biological pathways that offers an integrated environment for several mathematical representations ranging from stochastic to deterministic algorithms.

DiVinE is a general distributed verification environment meant to support the development of distributed enumerative model checking algorithms including probabilistic analysis features used for biological systems analysis [40].

StochKit [41] is a C++ stochastic simulation framework. Among other methods, it implements the Gillespie algorithm and in its second version it targets multicore platforms, it is therefore similar to our work. It does not implement online trajectory reduction that is performed in a postprocessing phase. A first form of online reduction of simulation trajectories has been experimented within StochKit-FF [11], which is an extension of StochKit using the FastFlow runtime.

In [14] a parallel computing platform has been employed to simulate a large biochemical network in hundreds different cellular volumes using Gillespie stochastic simulation algorithm on multiple processors. Parallel computing techniques made it possible to run massive simulations in reasonable computational times. However, the analysis of the simulation results to characterize the intrinsic noise of the network has been done as a postprocessing step. We believe our parallelization framework could further improve those kinds of analyses.

Hy3S software package [13] that includes hybrid stochastic simulation algorithms and SRSim [42] that performs rule-based spatial modeling are both embarrassingly parallelized by way of the MPI (message passing interface) library. In this case, high latencies and communication connection problems of the computing clusters could decrease the speed efficiency.

An efficient parallelization of Gillespie's SSA on GPGPU has been presented by Li and Petzold [43], where parallelization is obtained by distributing the computation of each trajectory to a distinct unit. Online data analysis has not been addressed; segmentation of threads and online alignment of outputs seem difficult to achieve owing to the sharing restrictions imposed by GPGPU. StochSimGPU [44] exploits GPGPU for parallel stochastic simulations of biological systems. The tool allows computing averages and histograms of the molecular populations across the sampled realizations on the GPGPU. The tool leverages on a GPGPU-accelerated version of the MATLAB framewosrk that can be hardly compared in flexibility and performance with a C++ implementation. A GPGPU implementation of the CWC simulator is actually under development. In particular, GPGPU exploitation appears to be particularly suitable for the analysis of spatial models (see [45–48]).

A schematic comparison of the main features of the biological simulation tools cited above is reported in Table 2.

This paper does not provide computational comparisons with other systems. Actually, a comparative analysis of the performance of the presented framework with respect to other simulators would not be particularly informative for the following reasons: (1) the computational cost of the simulations presented in this paper is mainly dependent on the parameters of the output sampling (time needed to write on disk); (2) the performance of our system is partially affected by the computational cost of the run time statistics, which the other simulators do not provide (this accounts, e.g., of about 6% of the total simulation cost in the Neurospora example with 10^5^ samples); (3) CWC, presented in this paper in a simplified form for the sake of readability uses a pattern matching algorithm in a compartmentalised setting (this makes it more expressive but computationally more expensive when compared with stochastic simulators implementing the Gillespie's algorithm in a flat scenario); and (4) another feature of CWC is the use of complex functions for computing the rate of reactions. So, for example, the model for the analysis of circadian oscillations of Neurospora cannot be directly simulated by Gillespie's algorithm.

## 5. Conclusions

In this paper we focused on a methodology to accelerate the simulation of stochastic models and analysis of simulation results on multicore platforms. We advocate a fully parallel simulation-analysis workflow as way to accelerate the simulation of stochastic model, to improve the likely to detect unknown system behaviors and to shorten the model tuning process thus improving tool usability.

We applied our methodology on a simulator for the calculus of wrapped compartments (CWC), a formal framework for modeling biological systems and their stochastic behavior. Even if we focused here on the integration of the simulation/analysis phases we demonstrate its efficiency and effectiveness by modeling three simple but paradigmatic examples of biological systems which are representative of different system dynamics, that is, multistable and oscillatory. All considered cases are representative of the kind of behaviors for which stochastic simulations have an expressivity edge onto classic ODEs. The ability of detecting interesting but unknown system behaviors is enhanced by the possibility of plugging in the workflow a set of user-defined statistic and mining tools that can be executed in parallel and while model simulation is still ongoing. In this respect our approach is, as far as we know, completely new.

Concerning the parallelization problem, we propose a fully parallel simulation-analysis tool that copes with several challenging problems, inter alia: close to ideal speedup and efficiency coupled with low code development effort; capability to manage high data throughput thus enhancing the precision of simulated models; and interactivity and reduced data size produced with respect to classic simulation-analysis sequential execution.

Many of these results are achieved by means of the FastFlow programming framework that provides a high-level of abstraction parallel programming methodology that exempt the programmer from direct management of synchronization and orchestration of concurrent activities. FastFlow provides low synchronization overhead and performance predictability, which makes it possible to design and tune a complex autobalancing, fully online simulation-analysis workflow that can produce data at a very high frequency and filter them in memory before being stored out-of-core avoiding the disk bottleneck.

The FastFlow framework and the CWC simulation-analysis workflow are open source software under LGPL license [23, 49].

## Figures and Tables

**Table 1:** Performance on 1200 simulation instances of the Neurospora model (Intel 32 core platform).

Single trajectory information		Overall data (20 sim eng, 3 stat eng)	
Number of samples	Interarrival time	Throughput	Output size
10^4^	25.86 *μ*s	2.70 MB/s	82.40 MB
10^5^	2.78 *μ*s	28.59 MB/s	823.98 MB
10^6^	232.68 ns	303.86 MB/s	8.24 GB

**Table 2:** Biological simulation tools comparison.

Tool	Calculus	Simulation schema	Parallelism	Data analysis
SCWC	CWC	Gillespie	FastFlow	Online statistics
SPiM	*π*-calculus	Gillespie	None	None
Dizzy	Reaction model	Gillespie, Gibson-Bruck, Tau-Leap, ODE	None	None
BioPEPA	Process algebra	ODE, Gillespie	None	None
Cellware	Reaction model	Gillespie, Gibson-Bruck, ODE	None	None
DiVinE	Model checker	ODE	MPI	None
StochKit	Reaction model	Gillespie, Tau-leaping	MPI	Postprocessing
StochKit2	Reaction model	Gillespie, Tau-leaping	Multithread	Postprocessing
StochKit-FF	Reaction model	Gillespie, Tau-leaping	FastFlow	Online statistics
Hy3S	Reaction model	Gibson-Bruck, Hybrid	MPI	Postprocessing
Li and Petzold's	Reaction model	Gillespie	GPGPU	None
StochSimGPU	Reaction model	Gillespie, Gibson-Bruck, Li	GPGPU	Postprocessing
